# Kinetics of the mechanochemical transformations in the “glycine - oxalic acid dihydrate” system revisited: The role of water

**DOI:** 10.3389/fchem.2025.1540129

**Published:** 2025-03-25

**Authors:** Evgeniy Losev, Polina Kalinina, Artem Golomolzin, Viktoria Kolesnikova, Elena Boldyreva

**Affiliations:** ^1^ V.S. Sobolev Institute of Geology and Mineralogy SB RAS, Novosibirsk, Russia; ^2^ Novosibirsk State University, Novosibirsk, Russia; ^3^ G.K. Boreskov Institute of Catalysis SB RAS, Novosibirsk, Russia; ^4^ V.V. Voevodsky Institute of Chemical Kinetics and Combustion, Novosibirsk, Russia; ^5^ Synchrotron Radiation Facility SKIF, G.K. Boreskov Institute of Catalysis SB RAS, Kol’tsovo, Russia

**Keywords:** mechanochemical synthesis, kinetics, ball milling, LAG, role of water in ball milling, choice of instrument, induction period of a mechanochemical transformation

## Abstract

**Introduction: **Kinetics of the mechanochemical transformations in the “glycine–oxalic acid dihydrate” system were revisited, in order to compare the results obtained for ball milling of the same reactants in different ball-milling devices.**Methods: **The results obtained in a commercial vibrational mill NARVA Vibrator DDR-GM9458 (ex situ study, this work) were compared with the previously published studies: ex situ in a home-made restricted-impact device and in situ in a Retsch MM400 vibrational mill.**Results: **We studied the effect of various factors on the mechanochemical transformations in this system under different conditions, such as the air humidity, the effect of the frequency of mechanical pulses on the existence of the induction period, the effect of the starting glycine polymorph on the duration of the induction period in case of a high-frequency vibrational ball milling, or the formation of G2O and GO as two competing products, the former dominating at the early stage of treatment as a “kinetic”, faster crystallizing phase, and the latter formed as the only final thermodynamically stable product after a prolonged treatment.**Discussion: **The abovementioned results were interpreted consistently considering the possibility that water released from oxalic acid crystal hydrate may have a significant effect on the mechanochemical transformations, even though it does not enter crystal structures of bis-glycinium oxalate (G2O) and glycinium semioxalate (GO) products.

## 1 Introduction

Mechanochemical transformations attract much attention. They often give products that cannot be obtained otherwise, and have several advantages as compared to thermal transformations: high yields and selectivity, short transformation time, no need to use large amounts of water or organic solvents (Boldyreva, 2013; Boldyreva, 2023; Michalchuk et al., 2021). In order to control mechanochemical transformations, one needs to take into account much more parameters than for a thermal one (Michalchuk et al., 2021). These parameters include the details of the mechanical treatment protocol, the presence (intentional or not) of additional compounds, the characteristics of the starting sample.

Kinetics of a mechanochemical transformation is a complex interplay of the kinetics of the chemical stages and the macrokinetics (mass and heat transfer) in the reaction jar (Boldyreva, 2023). Therefore, a kinetic study in mechanochemistry is a challenge. Various instrumental techniques are used to follow the changes in chemical and phase composition of mechanochemical transformations (Colacino et al., 2018).

Recently, *in situ* methods became increasingly popular. They make it possible to study a mechanochemical transformation at the intermediate time moments without having interrupted the process and opening the reaction jar. The samples can be analysed using a plethora of instrumental techniques, based on using various analytical methods, such as diffraction of synchrotron radiation (Michalchuk and Emmerling, 2022; Batzdorf et al., 2015; Martinetto et al., 2017; Friščić et al., 2013; Lampronti et al., 2021; Julien and Friščić, 2022), Raman spectroscopy (Batzdorf et al., 2015; Julien et al., 2017; Lukin et al., 2019; Lukin et al., 2021; Gracin et al., 2014), solid-state NMR spectroscopy (Mandala et al., 2014; Schiffmann et al., 2020; Xu et al., 2017; Gupta et al., 2014), terahertz spectroscopy (Lien Nguyen et al., 2007), fluorescence spectroscopy (Schramm et al., 2020; Julien et al., 2021), XANES (de Oliveira et al., 2020), often complemented by the control of the evolution of temperature and pressure in the jar (Oghenevweta et al., 2018; Tschakarov et al., 1982; Takacs, 2002; Han et al., 2021; Chen and Williams, 1996).

Obvious advantage of the *in situ* approach is that the measurements do not interfere with the process. At the same time, the *in situ* measurements are also not free of various weak points. The part of the sample that is accessible for the analysis may be not representative for the whole bulk (Michalchuk et al., 2014), a reactant or an intermediate product may disappear from the “view field” not because it has really transformed into another phase, but because of caking, sticking to the jar walls or the milling bodies (Michalchuk et al., 2017). These problems are attempted to overcome, or at least to reduce, either computationally (Michalchuk et al., 2017), or by modifying the construction of the mechanical activator and the mechanoreactors (Ban et al., 2017; Lampronti et al., 2021; Boldyreva, 2022). The experimental data collected not from a free sample, but in a jar can be of poorer quality because radiation used for the analysis has to penetrate through the jar walls. In order to make the walls more transparent for radiation, polymer materials are often used to manufacture the whole jar (Michalchuk and Emmerling, 2022; Batzdorf et al., 2015; Martinetto et al., 2017; Friščić et al., 2013; Lampronti et al., 2021; Mazzeo et al., 2022; Wilke et al., 2016), or a jar window (Rathmann et al., 2021). The change in the jar material can itself have a significant effect on the mechanochemical transformation–not only on its rate, but also on the phases formed as intermediate and final products (Germann et al., 2020; Losev et al., 2022; Chatziadi et al., 2022; Linberg et al., 2022). Last but not least, the *in situ* techniques are far less easily accessible for researchers than the *ex situ* studies of the mechanochemical transformations.

A common way to follow mechanochemical transformations in most laboratories and in industry is to analyse *ex situ* the reactant(s) before putting them into a device for mechanical treatment and then the product(s) that are extracted at an intermediate stage, or after the treatment has been completed. This seemingly simple approach, however, can give different results depending on its protocol (Michalchuk et al., 2014). For kinetic studies sampling is used, with two possible options. One option is that mechanical treatment is interrupted from time to time, the mechanical device is opened, and a part of the sample is taken for the analysis by one of the chemical or instrumental techniques, after which mechanical treatment is resumed. Another option is that the whole kinetic curve is “collected” from different samples, each of them being treated mechanically continuously, without interruptions and opening the jar, until a certain time, so that different points at the same kinetic curve correspond to different samples (Losev et al., 2022). The results may be different in the two cases. Continuous mechanical treatment during a certain time *t* can give different results from those obtained when treatment during shorter time intervals *t*_*1*_ *, t*_*2*_, etc. is alternated with periods when treatment is stopped for time intervals *t*_*1s*_, *t*_*2s*_, etc*.* (Boldyreva, 2023; Michalchuk et al., 2014; Boldyrev, 1996; Boldyrev, 1972; Boldyrev, 2006; Linberg et al., 2023; Belenguer et al., 2014; Schmidt et al., 2012; Howard et al., 2018; Kaneniwa and Otsuka, 1985; Tumanov et al., 2011). For some devices, like an extruder, the opening is not needed to probe the sample at an intermediate stage of mechanical treatment. However, even if the mechanochemical device is not opened, interruption of treatment can result, for example, in the cooling of the sample, relaxation of mechanical stress, and also in letting the system undergo a transformation that is thermodynamically necessary, but is kinetically hindered, and therefore does not require any further mechanical treatment after the first nuclei of the product phase(s) have been obtained mechanically and can now grow spontaneously (Boldyreva, 2023). Opening of a mechanical device is even more disturbing for the transformation. The contact to the atmosphere outside the reactor may have an effect on the humidity of the sample and as a consequence – on the outcome of the transformation (Michalchuk et al., 2014; Tumanov et al., 2017). The time separating the moment, when mechanical treatment has been stopped and the sample extracted, from the time when it has been analyzed in an *ex situ* experiment, can be sufficient for the sample to undergo significant changes (a transformation from amorphous state into a crystalline phase, recrystallization of smaller particles into larger ones, chemical transformations (Boldyreva, 2023). The probability that this in fact happens increases, if a probe needs to be preliminary treated before the analysis [ground, mixed with a diluting additive, which is supposed to be inert and non-interacting, but turns out to be not “inert” after a more careful study (Michalchuk et al., 2021; Bouvart et al., 2018)].

Another problem is related to the sampling procedure. One can either analyse all the sample in the reaction jar, or a part of it. In the former case, if more than one phase is detected in the sample, an open question is how these phases were distributed in the jar. This distribution is not necessarily homogeneous and may depend strongly on the shape of the reaction jar (spherical as in NARVA Vibrator DDR-GM9458, cylindrical as in Retsch MM400, etc.), the type of device and the treatment parameters (energy of ball impacts, vibrational frequency, etc.). Sampling from different parts of the jar may reveal different phases, since the conditions of treatment are different at different jar sites (Michalchuk et al., 2014). Quite often, mechanical treatment is accompanied by sample caking. In this case, the treatment is interrupted, the sample is extracted from the reactor, ground, put back into the reactor, and then the treatment is resumed. These actions may also change the outcome of the treatment–change the product(s), not merely the kinetics of the transformation (Michalchuk et al., 2014). One of the reasons for this is related to different exposure of the sample to humid air or to the fluid present in the solid sample (added on purpose, released on grinding from a crystal solvate, or formed in the course of a mechanochemical reaction between an acid and a base).

When kinetics of a mechanochemical transformation is analyzed, one often considers the results of treatment in one mechanical device, the choice of which may be arbitrary. One discusses the effects of various additives [liquids (Ying et al., 2021; Karki et al., 2007; Mukherjee et al., 2018; Hasa et al., 2015), polymers (Hasa et al., 2015; Friščić et al., 2010; Hasa et al., 2016), inorganic dilutants (Tsuzuki and McCormick, 1997), etc.], and also sometimes the changes resulting from different protocols of treatment using the same device (intensity, alternating treatment with relaxation, continuous/interrupted treatment, ball size, ball mass, balls to sample ratio, etc.) (Michalchuk et al., 2019). Studies comparing the outcome of mechanical treatment in different devices are much more rare and are focused on very different types of treatment (in an extruder, a ball mill, or a resonant acoustic mixer; in a low-energy vibrational mill and in a high-energy planetary ball mill) (Michalchuk et al., 2021). Comparing the course of mechanochemical transformations in different devices is important for practical purposes, in order to design and optimize technologies giving reproducible results, for scaling-up the processes. Such a comparison may be also helpful to get a better insight into the mechanisms of the mechanochemical transformations.

In the present study we revisited mechanochemical transformations in the system “glycine + oxalic acid dihydrate”. We compared the kinetics of the mechanochemical transformations when treating the same starting reactants in a vibrational mill–NARVA Vibrator DDR-GM9458 (this work) with the kinetics studied previously by *ex situ* technique using a model device with a falling load (Tumanov et al., 2011), and *in situ* at a synchrotron source using a vibrational Retsch mill MM400, also with a single ball (Michalchuk et al., 2017).

## 2 Experimental

### 2.1 Materials

**FIGURE 1:**
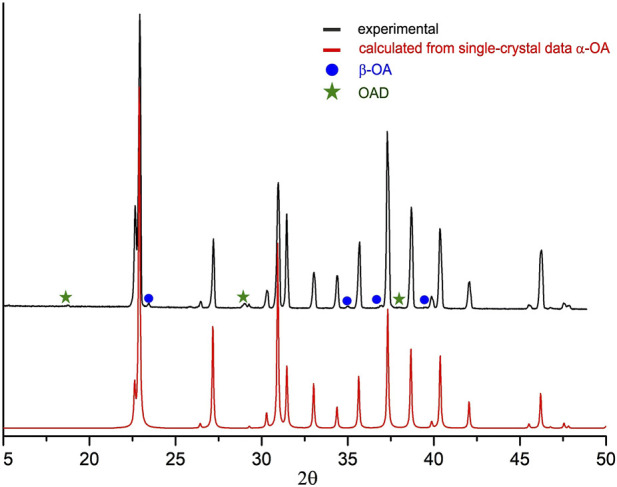
Powder X-ray diffraction pattern of dehydrated oxalic acid obtained in this work (above) and the one calculated based on the single-crystal X-ray diffraction data reported in (Thalladi et al., 2000).

Oxalic acid dihydrate (OAD) (Reakhim, 98%) and α-glycine (Sigma-Aldrich, 98%) were used as purchased. γ-Glycine was obtained from α-glycine (Sigma, 98%) by exposure in wet ammoniac vapor during a long time as described in (Boldyreva et al., 2003). Anhydrous oxalic acid (OA) was obtained by heating the OAD on the hot plate at 145°С for 3 h. Analysis of the powder diffraction pattern of the heated powder showed the presence of the anhydrous α-OA as the main product with an additional small amount of β-OA and trace amounts of OAD (Figure 1). OAD might be difficult to remove by heating or it might be formed due to the sorption of moisture from the air when powder is cooled. However, those impurities did not affect mechanochemical experiment. *Bis*-glycinium oxalate (G_2_O) was obtained by slow evaporation of a water solution of α-glycine and OAD in a molar ratio of 2:1 respectively.

### 2.2 Equipment and experimental scheme

The rate of a mechanochemical process at the initial stage is well-known to depend directly on the number and the area of contacts between particles (Boldyrev, 2006), therefore, before mixing and mechanical treatment the powders of all reagents were сlassified using Retsch sieves with cell diameters of 400 μm and 500 μm, so that the samples with particle sizes of only 400–500 μm were used for experiments. Samples were weighed using an OHAUS Analytical Plus analytical balance. Each mixture of reagents was mixed mechanically in a cylindrical container with one rotation axis for 1 minute to achieve a certain universal degree of mixing homogeneity before mechanical treatment. During described procedures no reaction was observed, which was controlled by Powder X-Ray Diffraction (PXRD).

**FIGURE 2:**
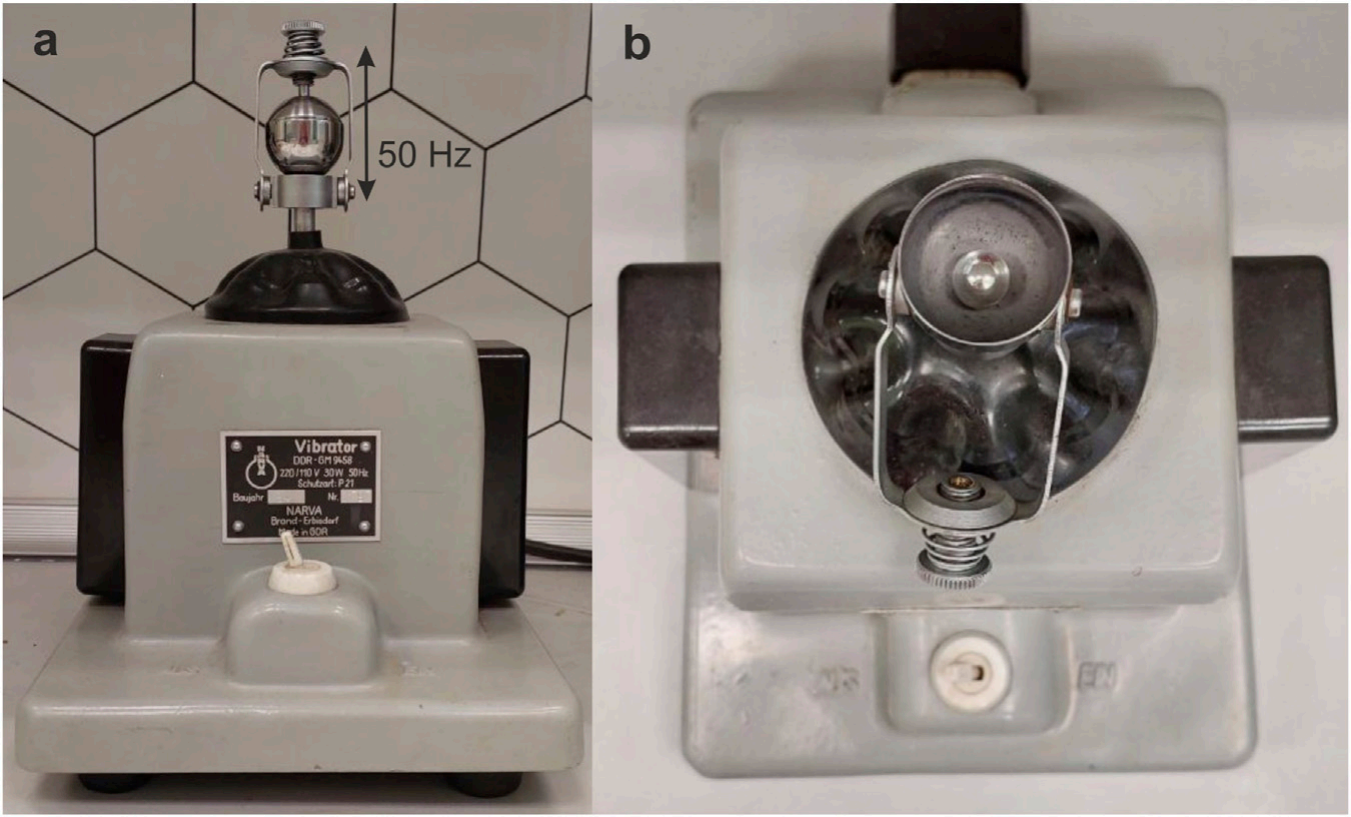
NARVA Vibrator DDR-GM9458 vibrational mill. **(a)** view from the side with a closed milling jar; **(b)** view from the top, steel ball is inside the jar.

Mechanical treatment was performed using a vibrational mill NARVA Vibrator DDR-GM9458 with a fixed processing frequency of 50 Hz (Figure 2), time of treatment was monitored by a stopwatch. Before treatment the insides of a steel milling jar, the surface of a grinding body (steel ball with a diameter of 9.5 mm and mass of 3.5 g) and tweezers, which were used for placing a ball into the jar were treated with ethanol and dried thoroughly in order to get rid of any contamination. Most experiments were carried out at a relative air humidity of 35 or 50%–60%. We did not observe any noticeable warming of the milling jar during the short treatment time that was needed to complete the transformations (not exceeding 3 min).

Phase composition after mechanical treatment was determined by powder X-ray diffraction using a multifunctional X-Ray diffractometer STOE STADI MP (Cu Kα1 radiation, λ = 1.54060Å, curved Ge (111) monochromator and a semiconductor linear detector Dectris MYTHEN 1K in a transmission geometry). The recording time of one diffraction pattern in the 2θ range from 5° to 60° was about 40 min. Full-profile analysis of diffraction patterns was carried out using the Rietveld method implemented in the GSAS-II program (Toby and Von Dreele, 2013). (see also Supplementary Information file)

To conduct the series of experiments with different reagents sample powders were selected each time as follows: an equimolar mixture of A (particle size 400–500 μm) and B (particle size 400–500 μm) were taken in such ratios as to keep the total mass equal to 40 mg. Approximate masses of reagents are listed in Table 1.

**TABLE 1:** Approximate masses of reagents in different series of experiments.

Mixture of reagents	α-glycine + OAD		G_2_O + OAD		α-glycine + OA		G_2_O + OA	
Reagents A and B	α-glycine	OAD	G_2_O	OAD	α-glycine	OA	G_2_O	OA
Approximate mass, mg	15.4	25.2	27.9	12.7	18.4	21.6	28.4	17.5

A series of independent experiments with multiple samples of an equimolar mixture of glycine with oxalic acid dihydrate was used for each kinetic curve. For each sample, the mechanical treatment was stopped at a different fixed time of continuous mechanochemical treatment (60 s, 90 s, 95 s, 96 s, 97 s, 100 s, 105 s, etc. in the α-glycine + OAD experiment 30 s, 40 s, 60 s, 140 s, 210 s in the G_2_O + OAD experiment 35 s, 38 s, 40 s, 42 s, 50 s, 60 s, 65 s, 75 s, 90 s, 100 s, 110 s, 120 s, 135 s, 160 s, 180 s in the γ-glycine + OAD experiment, so that the treatment was interrupted and the jar opened only once. After mechanical treatment, the whole treated sample was placed quickly in the sample holder and analysed by PXRD (the time from the extraction of the sample and the start of a diffraction experiment not exceeding a few minutes). 40 mg of powder was a sufficient amount of powder to fill our sample holder. Trace amounts of powder were left mostly on the milling ball. For a comparison, we collected probes from different parts of the jar during the trial experiment, and they all had the same phase composition, proving that the sample was homogeneous.

## 3 Results and discussion

**FIGURE 3:**
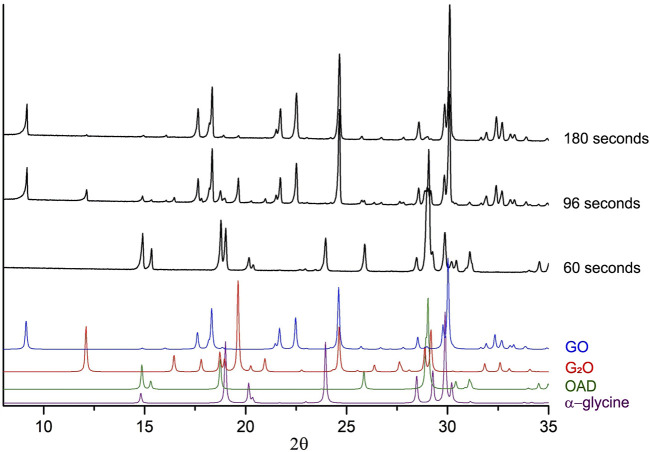
Experimental X-ray powder diffraction patterns collected in several independent “α-glycine + OAD” mechanochemical experiments. Each black diffraction pattern represents data obtained from a fresh sample after a different time of continuous mechanical treatment. Colour diffraction patterns represent patterns, calculated from single-crystal data for GO (Tumanov et al., 2010), G_2_O (Chitra and Choudhury, 2007), OAD (Wang et al., 1985) and α-glycine (Albrecht and Corey, 1939), which were added for a comparison.

**FIGURE 4:**
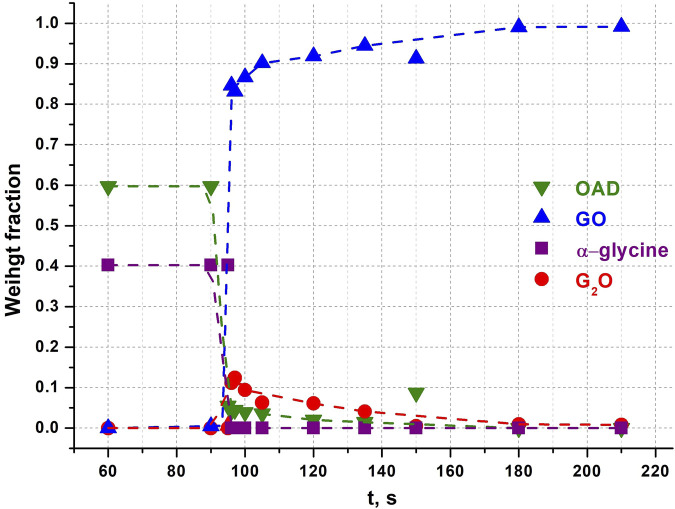
Weight fractions of different compounds obtained from Rietveld refinement of each diffraction pattern after different times of continuous mechanical activation in an α-glycine + OAD experiment. Points at 150 s seem like outlets. Dotted lines were added for clarity and visualisation of trends as guides to the eye.

Two different products can be formed on mechanical treatment of a powder mixture of glycine (Gly) and oxalic acid dihydrate (OAD): glycinium semioxalate (C_2_H_6_NO_2_^+^ ∙ C_2_HO_4_^−^), GO (Tumanov et al., 2010), as the final product, and *bis*-glycinium oxalate (2C_2_H_6_NO_2_^+^ ∙ C_2_O_4_^2-^), G_2_O (Chitra and Choudhury, 2007) as an intermediate phase (Michalchuk et al., 2017; Tumanov et al., 2011). Representative powder diffraction patterns collected in several selected experiments in this work are shown at Figure 3. We show as representative examples three powder diffraction patterns collected from the mixtures 1) during an induction period when no transformations have been observed yet (60 s), 2) at the moment when the transformations have started and an intermediate G_2_O product can be observed simultaneously with the final product GO (96 s), 3) final state after a complete transformation of the reactants into the final product, (180 s). The aim was to show the general view of the patterns, the baseline (smooth background, no evidence of amorphization), no significant broadening of the reflection profiles at all the stages of the transformation. The representative results of profile fitting that was used for the quantitative phase analysis of the samples are included as Supplementary Material. The corresponding kinetic curves for reagents and products are plotted at Figure 4. The kinetic curves had a pronounced induction period, lasting 95 s (∼1.5 min), after which the transformation started abruptly and the final product - glycinium semioxalate (GO) was formed to 80–90 wt.% in approximately 2 s. Immediately after the induction period another product, *bis*-glycinium oxalate (G_2_O), formed. During further mechanical treatment the G_2_O concentration decreased, whereas that of the GO increased. Glycinium semioxalate (GO) was the only final product of the transformation. The entire reaction mixture converted to GO after 250 s of continuous mechanical treatment.

**FIGURE 5:**
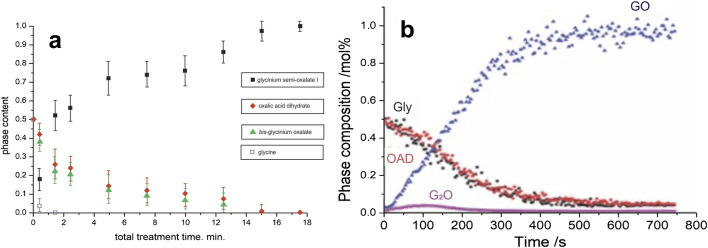
Kinetic curves for the mechanochemical transformations in the “glycine + OAD” system obtained in earlier work: **(a)** Ref. Tumanov et al. (2011), **(b)** Ref. Michalchuk et al. (2017). Reproduced with permission from the original sources.

Kinetics of the transformation in the “α-glycine + OAD” system observed in this work differed significantly from that measured in two previously reported experiments using different devices (Figure 5; Table 2).

**TABLE 2:** Comparison of the results of the mechanical treatment of “glycine–oxalic acid dihydrate” mixtures in three different devices.

	I (Tumanov et al., 2011)	II (Michalchuk et al., 2017)	III (this work)
Sample			
Polymorph	α-glycine	γ-glycine	α-glycine; γ-glycine
Particle size (initial)	100–150 μm, then agglomeration	400–500 μm	400–500 μm
Sample mass	100 mg	300 mg	15–30 mg
Mechanical device	a home-made restricted-impact instrument (Tumanov et al., 2011)	a Retsch MM400 vibrational mill (Michalchuk et al., 2017)	a NARVA Vibrator DDR-GM9458 vibrational mill (this work)
Mechanical treatment and data collection			
Preliminary mixing	Yes	Yes	Yes
Processing frequency	1.6 Hz (pulse 10–4 s; duration between pulses 0.67 s)	25–30 Hz	50 Hz
Sample analysis	*Ex situ*	*In situ*, data collected every 0.4 s	*Ex situ*
Interrupting the process	Only before taking the sample out	No	Only before taking the sample out
Opening the jar	Only before taking the sample out	No	Only before taking the sample out
Shape of the jar, jar material	A curved surface hit by a falling cylindrical load, stainless steel	Cylindrical, perspex	Spherical, stainless steel
Ball mass	Energy of an impact by a falling working body 22 mJ; 15.4 g weight was dropped from 17.5 cm	3.5 g	3.5 g
Ball diameter		7 mm	9.5 mm
Ball number		1	1
Part of the sample analyzed	All the sample	The sample in the synchrotron beam	All the sample
Air humidity	Not controlled	Not controlled	Controlled and varied from 35% to 60%
Results of kinetic studies			
Induction period	No	No	Yes 95 s (∼1.5 min)
Intermediate product	G_2_O	G_2_O	G_2_O
Final product	GO	GO	GO
Time before reaction completed	1200 s	720 s	250 s

### 3.1 Induction period

The main difference is that no pronounced induction period was observed in previous experiments. Induction period may indicate that some “invisible” processes in the system occur, before a transformation becomes observable. These processes may be quite different from each other. In principle, an induction period may be related to mixing of reactants improving their contacts (Lapshin et al., 2021), but this option is excluded in our experiments, since the reactants were already mixed carefully before being put into the reaction jar. Another option is that the particle size is reduced during the induction period, so that the transformation rate increases sharply only after the particles of reactants become small enough (Lampronti et al., 2021). This option is in principle possible, but it could not be supported experimentally, since no broadening of X-ray diffraction patterns was observed for the samples subjected to mechanical treatment. The diffraction patterns of the mixture of initial reagents at treatment times of 25, 40, 60 and 95 s are completely identical. At the same time, a noticeable broadening of X-ray diffraction patterns could be observed only if the size of particles was reduced from original 400–500 nm to 100. There was no evidence of disorder, a decrease in coherent scattering regions, or an increase in background intensity during the induction period.

The existence of the induction period is in general a common feature of many solid-state transformations and is usually related with reaction starting at selected “active sites” and then self-accelerating due to positive feed-back (autocatalysis) (Boldyrev et al., 1979). With the development of techniques for time-resolved *in situ* (TRIS) monitoring of mechanochemical reactions, there is mounting evidence that typical mechanochemical reactions also often have induction periods (Hutchings et al., 2017; Linberg et al., 2023). It was supposed to result from a need to mechanically activate the material prior to transformation (Linberg et al., 2023), so that there is no real conceptual “gap” between “preliminary mechanical activation” and a “mechanochemical reaction” (Boldyreva, 2023; Boldyrev, 1996).

Mechanical treatment in a vibrational ball mill is not truly continuous, but consists of repeated mechanical impacts. Various processes may occur either during a mechanical impact, or between the impacts (Boldyreva, 2023; Boldyrev, 1972; Boldyrev, 2006). They may be related to the phenomena occurring in the solid state (accumulation of defects, fragmentation, seeding of a new phase - usually during an impact; secondary rearrangement of defects, polymorphic transformation as a result of seed growing–already after an impact), or in a fluid state (dissolution, recrystallization, chemical reaction – already after an impact). For inorganic compounds, activation during the induction period is known to occur usually through the formation of defects in the solid. The periodic dynamic mechanical stresses imposed by ball milling drive the solid away from its thermodynamic equilibrium state into a metastable (activated) state (Boldyreva, 2023; Boldyrev, 1972; Boldyrev, 2006). For organic compounds other options may be more likely (Boldyreva, 2013). In a study of a mechanochemical Knoevenagel condensation of vanillin and barbituric acid, the origin of the induction period was found to be a feedback cycle involving both chemical and mechanical factors and non-obvious and dynamic rheological changes in the reaction mixture. During the reaction the physical form of the reaction mixture changed from a powder to a cohesive rubber-like state, and this resulted in the observed reaction rate increase after the induction period (Hutchings et al., 2017). In a recent study (Linberg et al., 2023), a very long (hours) induction period of the formation of the final product of a mechanochemical synthesis of 1: 1 cocrystal of carbamazepine and isonicotinamide was shown to be caused by a delayed mechanically driven solid-state polymorphic transformation of one polymorph of this cocrystal into another. In our case, however, no polymorphic transitions were detected neither for the reactants, nor for the final (GO) and intermediate (G_2_O) products.

In order to elucidate the nature of the processes that account for the induction period of a mechanochemical transformation, it is helpful to consider the effect of impact frequency on the induction period. A comparison of the results of mechanical treatment with different impact frequencies in this study and in the two previous ones (Michalchuk et al., 2017; Tumanov et al., 2011) (Table 2) may suggest that the processes that are important for the reaction between Gly and OAD take place between the impacts: induction period was observed, if the impact frequency was high (experiments in this work), i.e., there was not sufficient time between the impacts to let the processes that are important for the reaction trigger the reaction immediately.

One of the reactants, OAD, is a crystal hydrate. The mole amount of crystal water (2 mol of water per 1 mol of oxalic acid) is comparable with that of the oxalic acid and glycine that participate in the mechanochemical reaction to form an intermediate product with 2:1 glycine:oxalic acid ratio (G_2_O) and the final product with the 1:1 glycine:oxalic acid ratio (GO). It is known that such a significant amount of crystal water may have a significant effect on a mechanochemical transformation, comparable with that of liquid water added to the system in a liquid-assisted grinding (LAG) process (Losev and Boldyreva, 2014). We have supposed that water may also play an important role in the “Gly + OAD” system, even though the products are not crystal hydrates. If a mechanical impact helps to release water from OAD, then the molecular salts in the “glycine-oxalic acid” system may form not in the solid state, but in water solution. When the system is mechanically treated at high frequencies (50 Hz), the fluid phase has no sufficient time to be formed in between two subsequent mechanical pulses. Therefore, the reaction is not observed, until a certain critical amount of water molecules has been released that can facilitate the interaction between glycine and oxalic acid in a fluid, or a fluidized state. Subsequently, with continued application of mechanical load, the reaction proceeds like an avalanche with an almost complete transition of the reagents into the final GO product of the reaction in 2–3 s.

We have carried out additional experiments to test this hypothesis.

**FIGURE 6:**
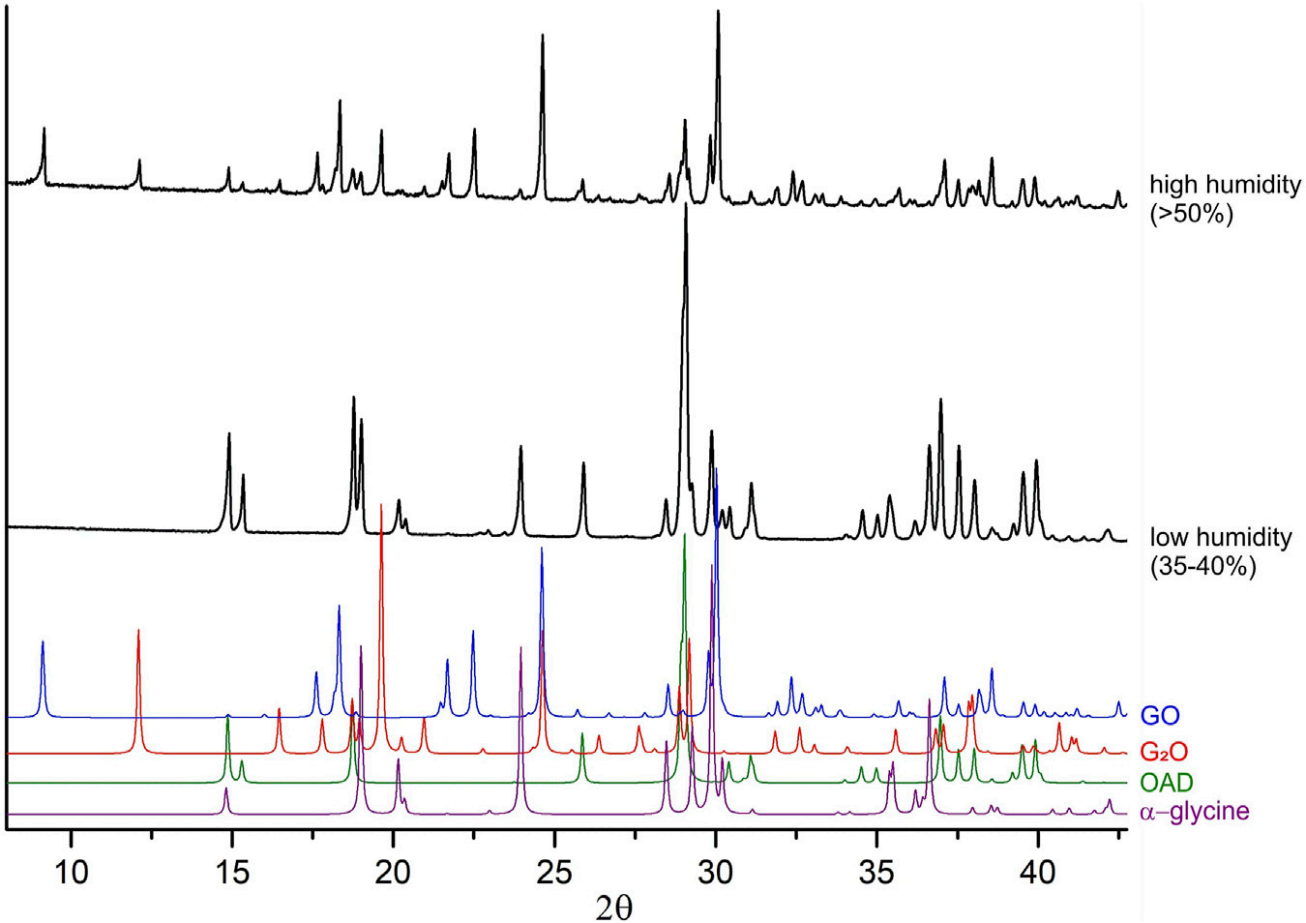
Experimental powder diffraction patterns for the mixtures “α-glycine + OAD” mechanically treated at different air humidity (different seasons). Black diffraction patterns represent data obtained after 60 s of mechanical treatment at high humidity (spring), and at low humidity (winter). Color diffraction patterns were calculated from single-crystal diffraction data for GO (Tumanov et al., 2010), G_2_O (Chitra and Choudhury, 2007), OAD (Wang et al., 1985) and α-glycine (Albrecht and Corey, 1939), for reference.

According to the previously published data, the treatment of a mixture of α-glycine with anhydrous oxalic acid does not result in any reaction (Losev et al., 2013), supporting the hypothesis of the key influence of the presence of water molecules in the system on the course of the mechanochemical reaction. In this work, ball milling of a mixture of α-glycine with anhydrous oxalic acid for 3 min also did not give any products. The reaction could be observed, however, if the air was humid. It is worth mentioning, that also the induction period of the reaction of Gly with OAD shortened with increasing air humidity: in spring (50–60 rel.% humidity) it was shorter as compared to the experiments performed in winter (about 35 rel.% humidity) (Figure 6). Such seasonable irreproducibility of mechanochemical reactions related to the effect of air humidity on the process has been documented earlier for other systems (Tumanov et al., 2017).

In the next series of experiments, mixtures of glycine and OAD were preliminary mechanically treated for 25 s and then stored in a closed jar. After 2 h of storage the mixture contained only the starting reagents (glycine and OAD), as was shown by X-ray powder diffraction. A complete conversion of the reagents into the final product, GO, was observed, when the storage period was increased to a week. This series of experiments supports the hypothesis that the reaction between Gly and OAD is initiated through the release of hydration water molecules from the OAD structure. Water facilitates this reaction if present as a fluid–sorbed from the air or formed on dehydration of OAD. One can suppose that it is the release of the sufficient amount of water that occurs during the induction period of the mechanochemical synthesis between the mechanical impacts. The impacts themselves destabilize the crystal structure of OAD and facilitate the dehydration. Remarkably, mechanical treatment of OAD alone does not give anhydrous oxalic acid in any detectable amount (Losev and Boldyreva, 2014; Losev et al., 2013). Apparently, an equilibrium between the hydrated and the anhydrous forms is shifted very much to the hydrated one, unless there is a chemical substance other than water, that can react with the anhydrous oxalic acid, to form a salt. In this work this “other substance” is glycine. One can also suppose that the rate of the water release depends on the number of defects in the crystal structure of OAD, which are formed at the stage of preliminary mechanical activation during the induction period.

### 3.2 Formation of G_2_O and GO

Another interesting feature of the mechanochemical transformations in the “Gly + OAD” system is that first G_2_O is formed, which then disappears from the reaction mixture to be substituted completely by the final product, GO. This sequence of transformations was observed in all the experiments, also in this work. The details of kinetics, however, differed from experiment to experiment, depending on the protocol of mechanical treatment. We tried to rationalize these observations taking into account the possible role of water in the process.

Analysis of the product composition at different times of mechanical treatment in this work shows the formation of *bis*-glycinium oxalate (G_2_O) in the system at a maximum concentration (about 12 wt.%) immediately after the end of the induction period (simultaneously with an increase in the content of the final GO product) and a subsequent drop in its content simultaneously with an increase in content of the final reaction product in the form of GO (Figure 4). This observation has a reasonable explanation from the point of view of the difference in aqueous solubility of α-glycine (∼0.25 g/mL in H_2_O at 25°C) and oxalic acid (∼0.12 g/mL in H_2_O at 25°C). The moment the transformation begins, the water released from crystalline OAD is more supersaturated with respect to glycine than with oxalic acid (the maximum possible volume of added water, obtained from the calculation of the complete decomposition of 25 mg of oxalic acid dihydrate, is 7.2 μL). A stoichiometric glycine predominance in a solution leads to partial crystallization and growth of G_2_O despite the equimolar composition of the initial mixture of reagents. Subsequently, the G_2_O formed initially reacts with the remaining oxalic acid molecules, presumably also with water solution involved, to form more GO.

The peak G_2_O concentration observed in this work is about 5 mol.% (or 12 wt.%), which is at the level of the G_2_O content obtained as a result of *in situ* experiments at the synchrotron radiation source at processing frequencies of 25–30 Hz (Michalchuk et al., 2017) and differs significantly from the G_2_O concentration determined in an *ex situ* experiment on a model setup at a processing frequency of 1.67 Hz (Tumanov et al., 2011). It was suggested (Michalchuk et al., 2017), that such a difference may be associated with the predominant formation of G_2_O in the end parts of the mill container, because the formation of G_2_O is facilitated by the direct impact, as well as by the difficulties of its detection when positioning the synchrotron radiation beam in the area of the walls (Michalchuk et al., 2017). This model also provides an explanation for the significant G_2_O content (about 40 mol%) at the initial processing times when using a model apparatus with a falling weight (Tumanov et al., 2011). The results obtained in the present work do not, however, support this hypothesis, since in the case of the NARVA Vibrator DDR-GM9458 mill the entire volume of the substance is subjected primarily to impacts due to the vertical direction of displacement of the jar and grinding body during operation. The results of this work can suggest that the initial content of G_2_O is likely to be determined by the frequency of treatment, namely by the relaxation time of the system between impact pulses. A longer relaxation time promotes the release of more crystal hydrate water, which leads to the crystallization predominantly of G_2_O due to the difference in solubility of glycine and OAD.

**FIGURE 7:**
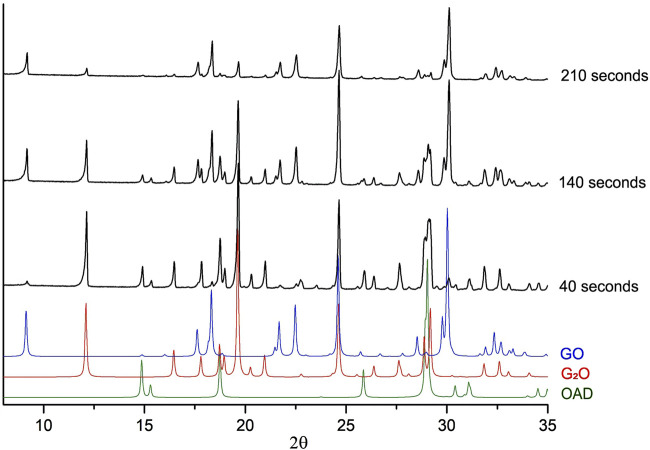
Experimental powder X-ray diffraction patterns of the mixture of initially G_2_O + OAD after mechanical treatment. Each black diffraction pattern represents data obtained after different times of mechanical activation. Colored diffraction patterns represent patterns, calculated from single-crystal diffraction data for GO (Tumanov et al., 2010), G_2_O (Chitra and Choudhury, 2007) and OAD (Wang et al., 1985), which were added for reference.

In order to gain more understanding of the kinetics of the accumulation and disappearance of G_2_O and GO in the “Gly + OAD” system on mechanical treatment, additional experiments were carried out. During those experiments a mixture of OAD with G_2_O was mechanically treated. G_2_O was obtained by crystallization from an aqueous solution at a molar ratio of glycine to oxalic acid of 2:1. Powder diffraction patterns of several chosen experiments are shown on Figure 7.

**FIGURE 8:**
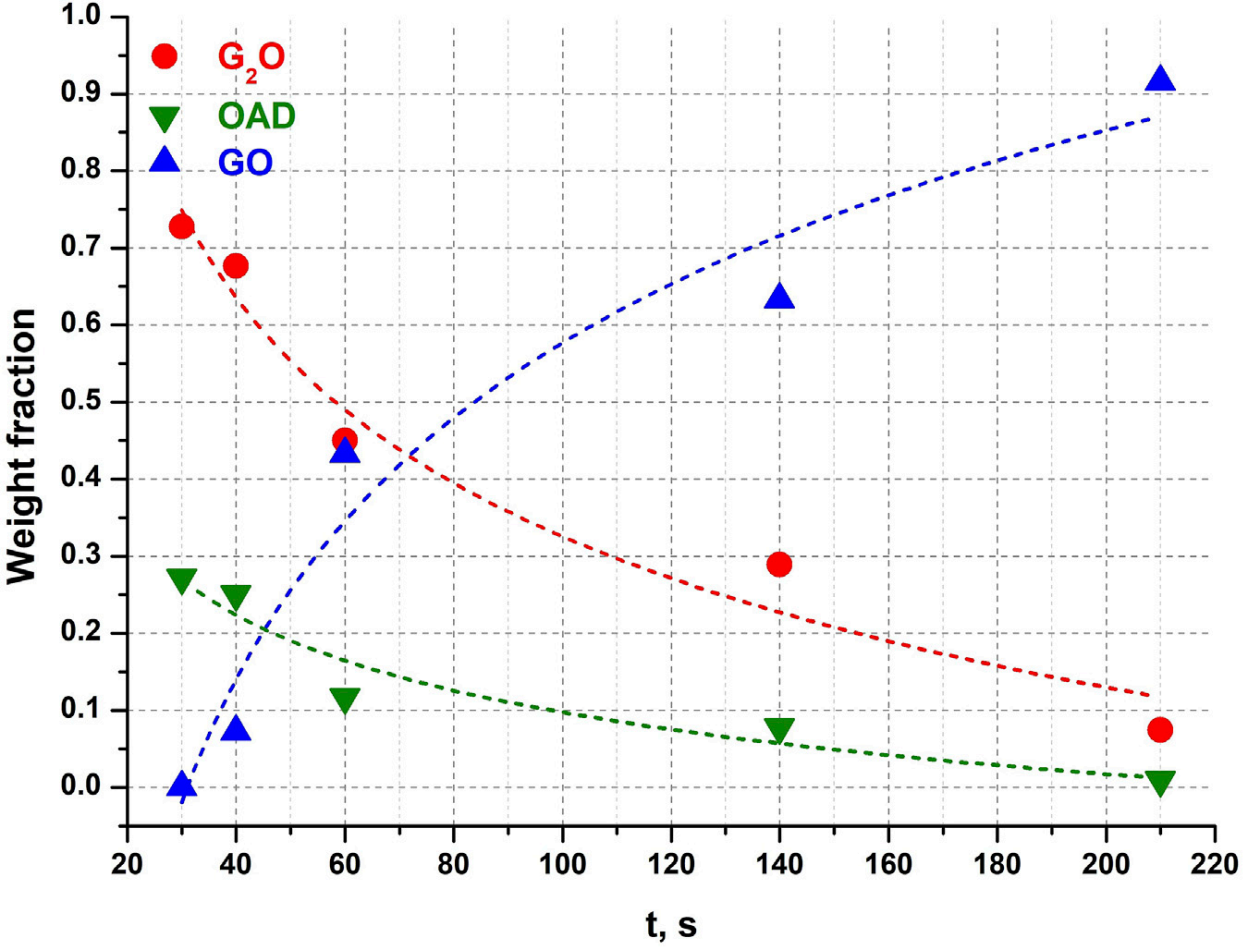
Weight fractions of different compounds obtained from Rietveld refinement of each diffraction pattern after different times of mechanical activation in a G_2_O + OAD experiment. Dotted lines are not real kinetic functions, they were added for clarity and visualization of trends.

Complete conversion of the reagents into the product (GO) took approximately the same total time as when OAD was milled in a mixture with α-Gly (Figure 4). However, the induction period was significantly shorter (about 35 s), as compared to 95 s when α-glycine was a starting reagent. The reaction progressed more smoothly, so that the amount of GO did not increase abruptly immediately after the induction period (Figure 8). One can relate a longer induction period of the mechanochemical reaction with α-Gly with a higher lattice energy of α-glycine as compared with G_2_O, that makes it more difficult to break the intermolecular hydrogen bonds and dissolve α-glycine in water as compared with its salt. Lattice energies often correlate with melting temperatures and solubility values (Perlovich, 2017; Perlovich, 2020). A comparison of the melting temperatures of α-glycine and G_2_O shows a difference of almost 100°C [250°C for α-glycine (Boldyreva et al., 2003; Perlovich et al., 2001) and 155°C for G_2_O (Chitra and Choudhury, 2007; Fleck and Petrosyan, 2014; Uma Devi et al., 2008)], which indicates a higher energy of intermolecular interactions, mainly hydrogen bonds, stabilizing the structure of glycine as compared to its molecular salt.

A question that remained unanswered in the previous studies of the mechanochemical reactions in the “Gly + OAD” system was if solid GO, the final product, forms from solid G_2_O as an intermediate phase resulting from a solid-state transformation (decomposition, elimination of half of glycine cations), or the two glycine salts with different stoichiometries are formed independently during parallel reactions, and G_2_O content decreases since this salt, first formed faster, then dissolves and recrystallizes into a more stable GO form. The second option requires the presence of water. In fact, no reaction between G_2_O and anhydrous oxalic acid could be observed in this work after a continuous mechanical treatment for 3 min. The G_2_O alone is stable with respect to mechanical treatment and did not transform into GO when ball-milled for 3.5 min, either dry, or in the presence of an equimolar amount of distilled water.

### 3.3 Reactions with α–and γ-glycine

**FIGURE 9:**
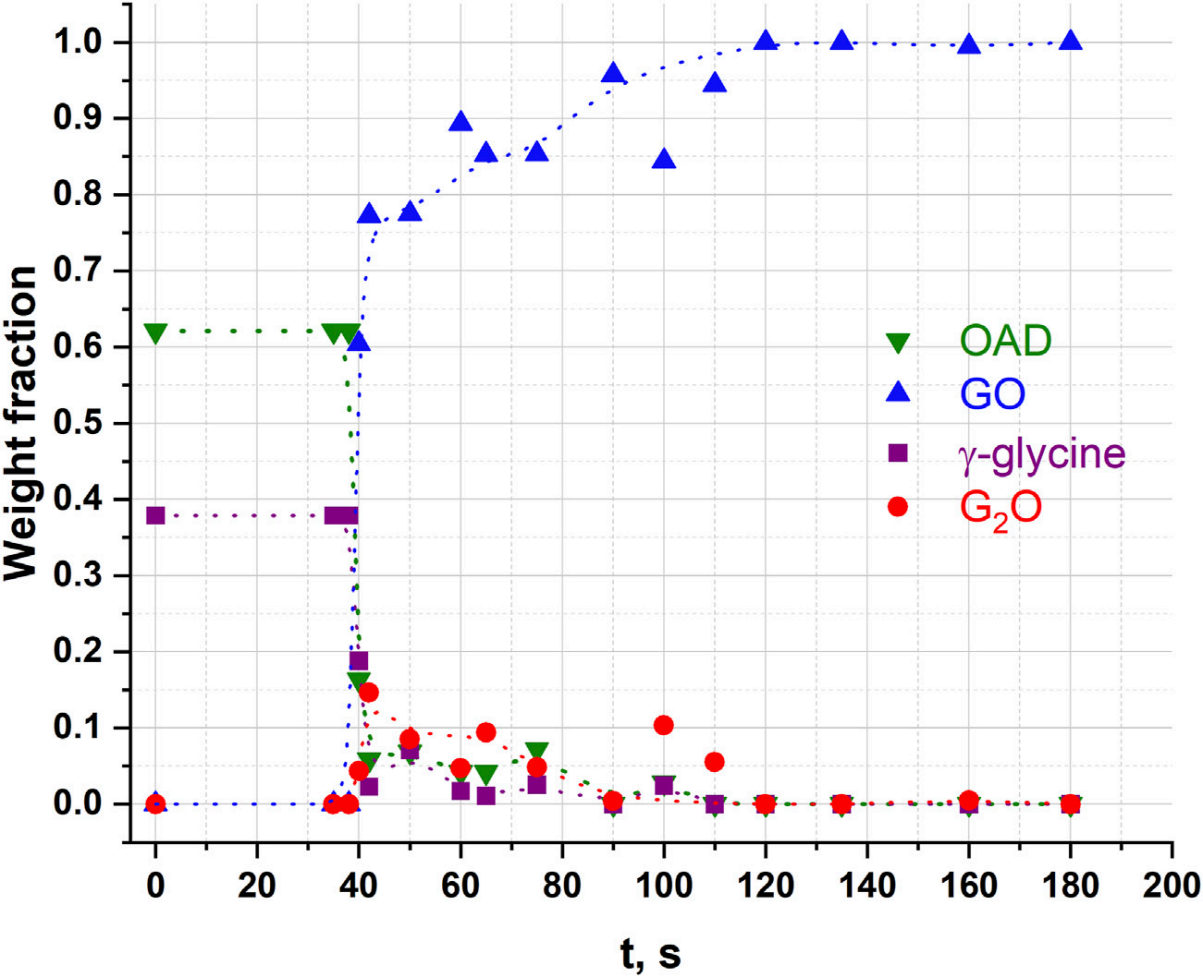
Weight fractions of different compounds obtained from Rietveld refinement of each diffraction pattern after different times of сontinuous mechanical activation in an γ-glycine + OAD experiment. Dotted lines were added for clarity and visualisation of trends as guides to the eye.

Glycine is prone to polymorphism (Boldyreva, 2021). In previous publications different polymorphs were treated in different mechanical activators (Table 1), and this complicates a comparison of the results. Therefore, in this work we have carried out a comparative study of α– and γ-glycine in a NARVA Vibrator DDR-GM9458 vibrational mill under identical conditions. Kinetics of the transformations with γ-glycine (Figure 9) was qualitatively similar to that with α–glycine (Figure 4): it also was characterized by a pronounced induction period, but this induction period was shorter, than in the case of the reaction with α–glycine. One can relate this difference with the difference in the interactions of the two glycine polymorphs with water, in particular, with the kinetics of their dissolution (Parks et al., 2017). The choice of the starting polymorph seemed to be less important for the existence of the induction period, than the choice of the instrument for mechanical treatment, in particular, of the frequency of mechanical impacts.

## 4 Conclusion

Mechanochemical synthesis of organic salts and cocrystals is becoming more and more common. In many cases a small amount of fluids is added to the powder mixture on purpose and such processes are termed Liquid Assisted Grinding (LAG). The role of liquid in such processes is well documented. At the same time, liquids can play a significant role also when not added specially. For example, fluids can be adsorbed from the air, or form on melting. Quite often, starting reactants are crystal hydrates (or solvates), and even if they are originally taken as dry solids, water is released on mechanical treatment. Importantly, the amount of water in this case is comparable with the content of the chemical species that form the product. Even if water does not enter the product structure, its role in the transformation may not be neglected. Mechanochemical transformations in the “glycine + oxalic acid system” can serve as a model for many other mechanochemical syntheses in which crystal hydrates are involved. Interestingly, mechanical treatment of oxalic acid dihydrate alone does not give any noticeable amount of anhydrous oxalic acid. If another component, namely, glycine is available to form a salt with oxalic acid, such a salt forms, i.e., water is released from oxalic acid crystal hydrate and may have a significant effect on the transformation, even if it does not enter this new structure. Considering this possibility, we could explain various facts related to the mechanochemical transformations in this system under different conditions. These conditions include air humidity and the effect of the frequency of mechanical pulses on the existence of the induction period. Additionally, we can examine the effect of the starting glycine polymorph on the duration of the induction period during high-frequency vibrational ball-milling. Furthermore, we can analyze the formation of bis-glycinium oxalate (G2O) and glycinium semioxalate (GO) as two competing products. The former, G2O, dominates at the early stage of treatment as a “kinetic,” faster-crystallizing phase. In contrast, GO is formed as the only final thermodynamically stable product after prolonged treatment

Obviously, there are many different factors that determine a course of a mechanochemical transformation. From Table 2 one can see, that many parameters were modified simultaneously in experiments described in the two earlier publications (Michalchuk et al., 2017; Tumanov et al., 2011), and in this work. Still, large variations in many different parameters, including changing the starting glycine polymorph, the jar material, the type of mechanical device, the sampling procedure, etc. did not lead to such a dramatic change in the kinetics as the appearance of induction period of the transformation. Only the increase in the treatment frequency to 50 Hz resulted in the appearance of the induction period. This makes us believe, that our hypothesis that the processes between the pulses playing a crucial role in the mechanochemical transformations in “glycine–oxalic acid dihydrate” system is correct. Various experiments with water (added specially, soaked from humid air, formed on dehydration of a reactant), as well as observations of the transformations in the solid mixtures of reactants after preliminary mechanical treatment support also the hypothesis that at least some of the important stages of the mechanochemical transformations in this system proceed in the fluid state, namely, in the aqueous solution or at the solid-liquid interface.

The work is important for the understanding of the mechanisms of mechanochemical transformations involving molecular crystals and their crystal solvates, including pharmaceutically active substances. The presence of a fluid in general, and water, in particular, may play a key role in such transformations. It can be rationalized by a systematic comparison of the results of treatment using different instruments, as well as by modifying the starting reactants.

## Data Availability

The raw data supporting the conclusions of this article will be made available by the authors, without undue reservation.
