# Supplementary material for: Kinetics of the mechanochemical transformations in the “glycine - oxalic acid dihydrate” system revisited: The role of water
Source: Front Chem. 2025 Mar 25;13:1540129. doi: 10.3389/fchem.2025.1540129 (PMC11975923; doi:10.3389/fchem.2025.1540129)
Supplement: Supplementary file 1 [file DataSheet1.docx]

**Supplementary Information for the manuscript by Evgeniy Losev, Polina Kalinina, Artem Golomolzin, Viktoria Kolesnikova and Elena Boldyreva “Kinetics of the mechanochemical transformations in the “glycine – oxalic acid dihydrate” system revisited: the role of water”.**

Examples of Rietveld refinement of the powder X-ray diffraction patterns used for quantitative phase analysis.


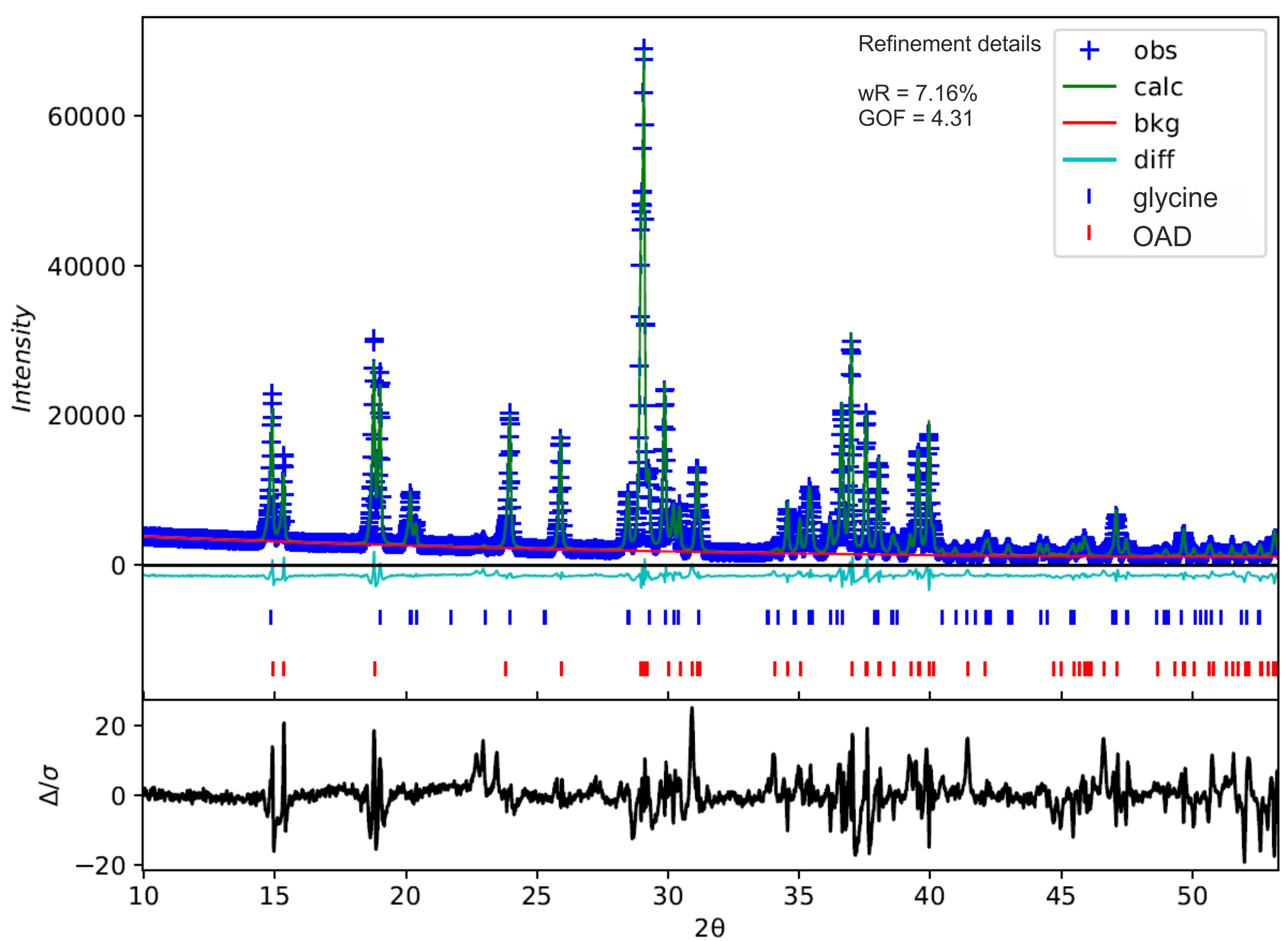


Figure S1. Profile fitting of the powder diffraction pattern: α-glycine + OAD mixture, treated for 60 s (Figures 3 and 6 (low humidity) in the main text)


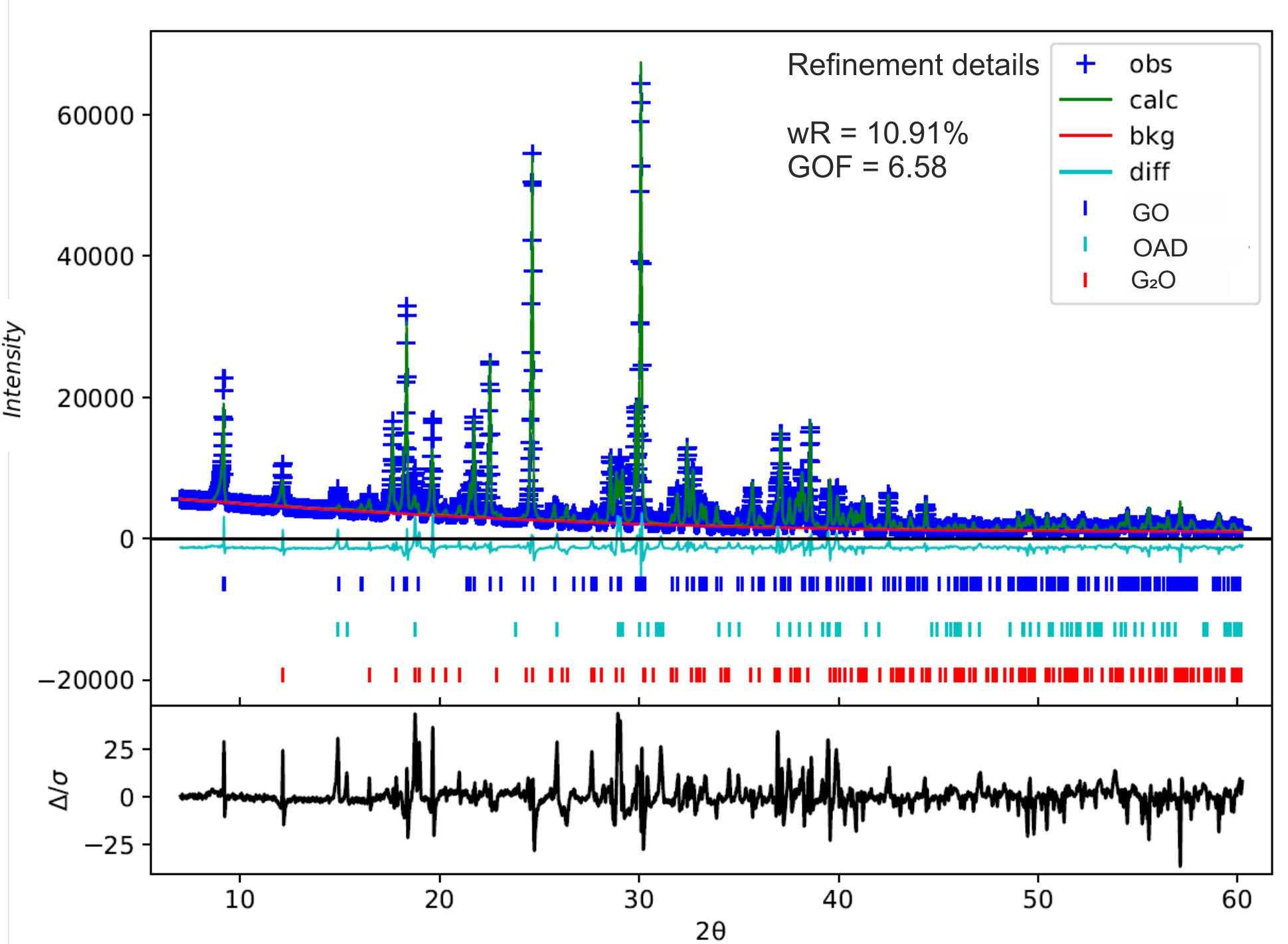


Figure S2. Profile fitting of the powder diffraction pattern: α-glycine + OAD mixture, treated for 96 s (Figure 3 in the main text)


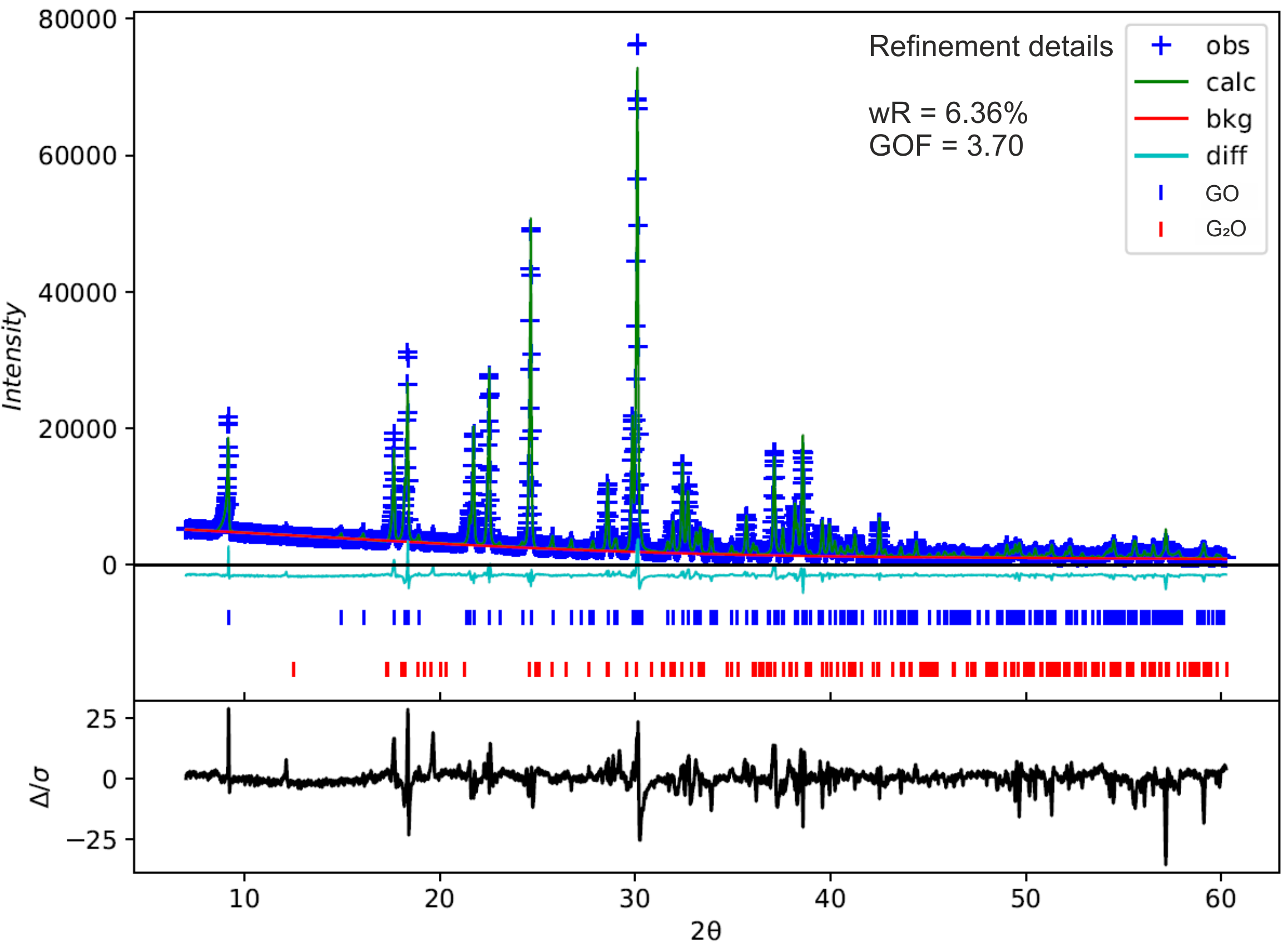


Figure S3. Profile fitting of the powder diffraction pattern: α-glycine + OAD mixture, treated for 180 s (Figure 3 in the main text)


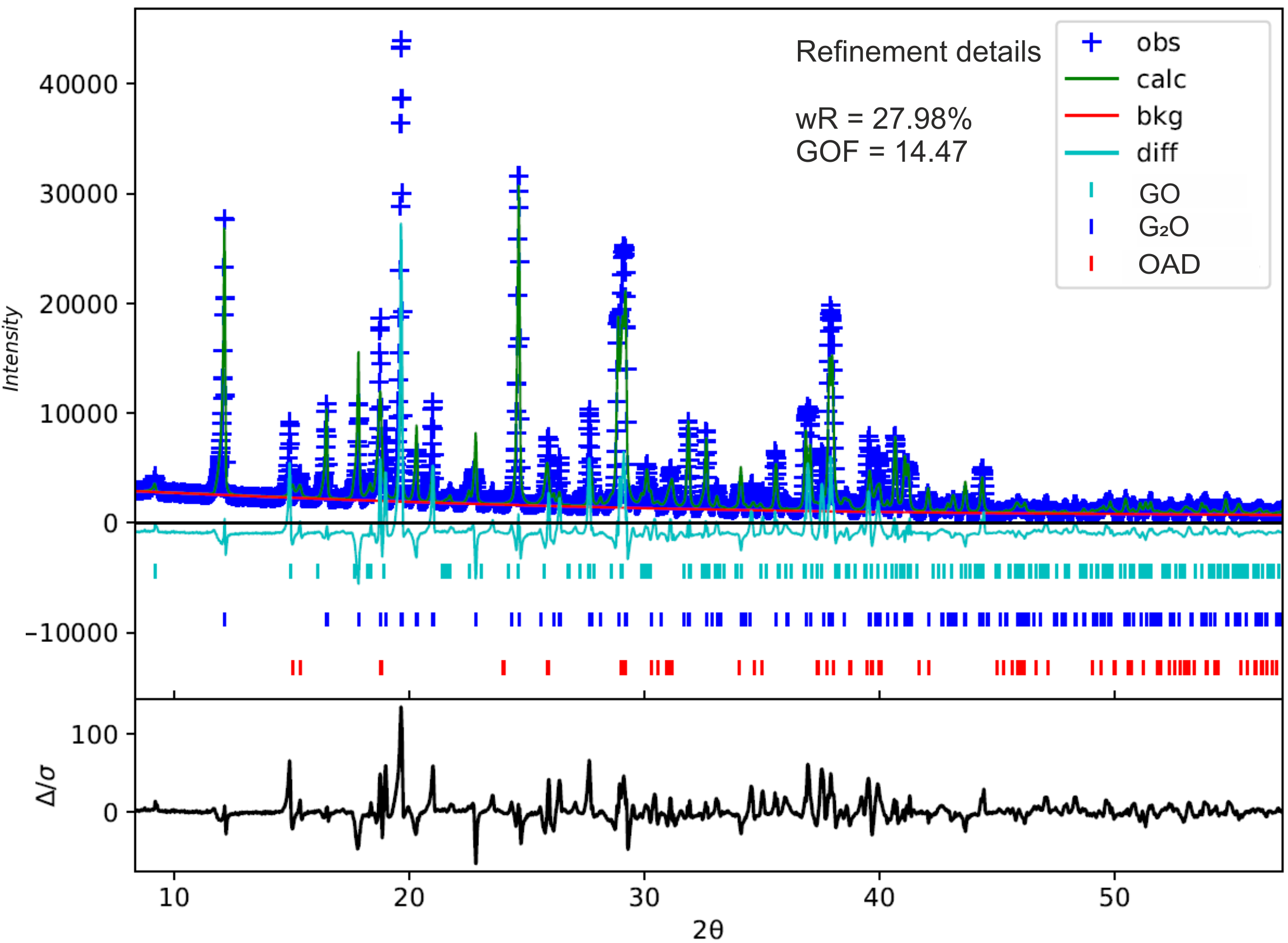


Figure S4. Profile fitting of the powder diffraction pattern: G_2_O + OAD mixture, treated for 40 s (Figure 7 in the main text)


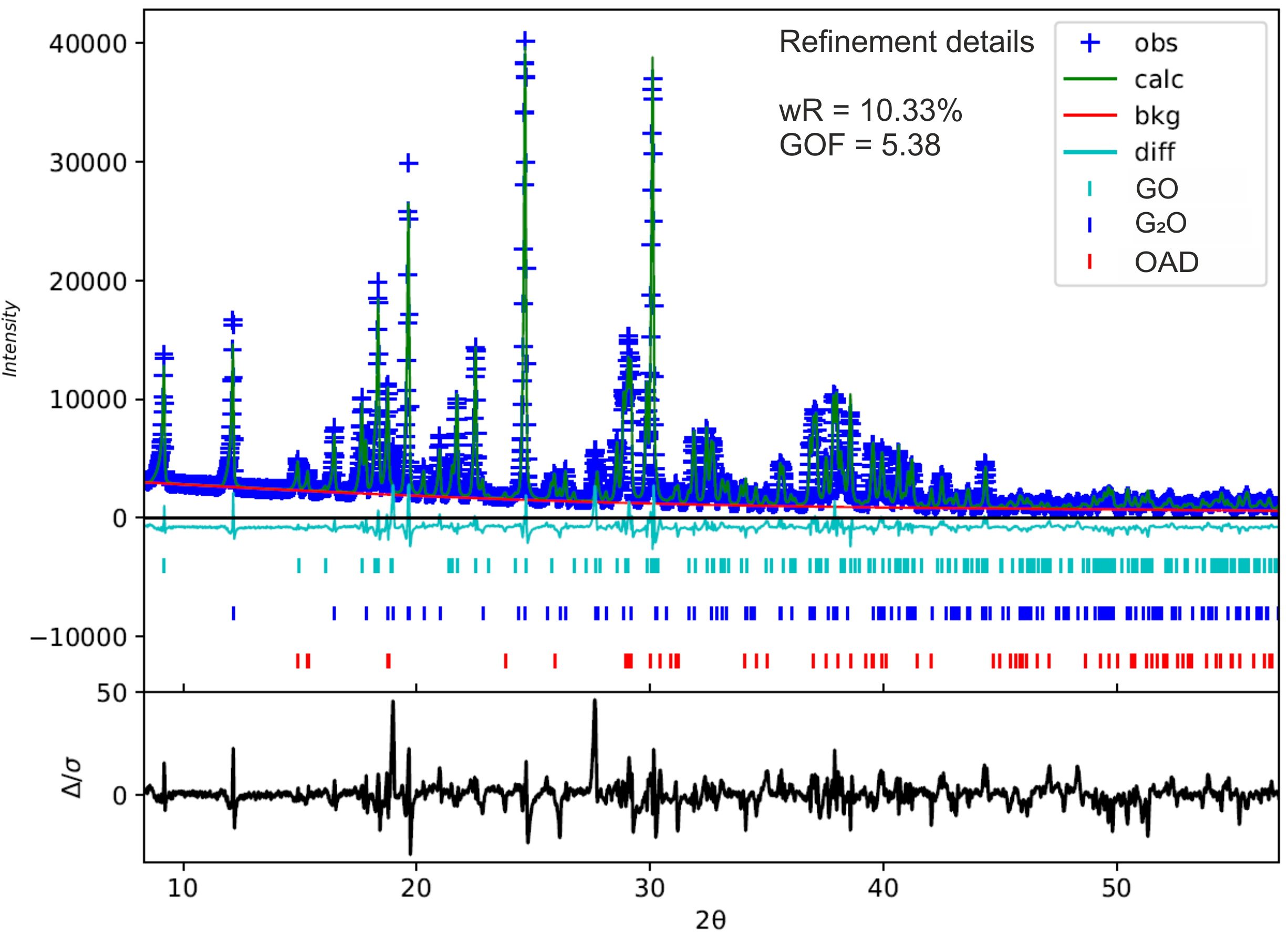


Figure S5. Profile fitting of the powder diffraction pattern: G_2_O + OAD mixture, treated for 140 s (Figure 7 in the main text)


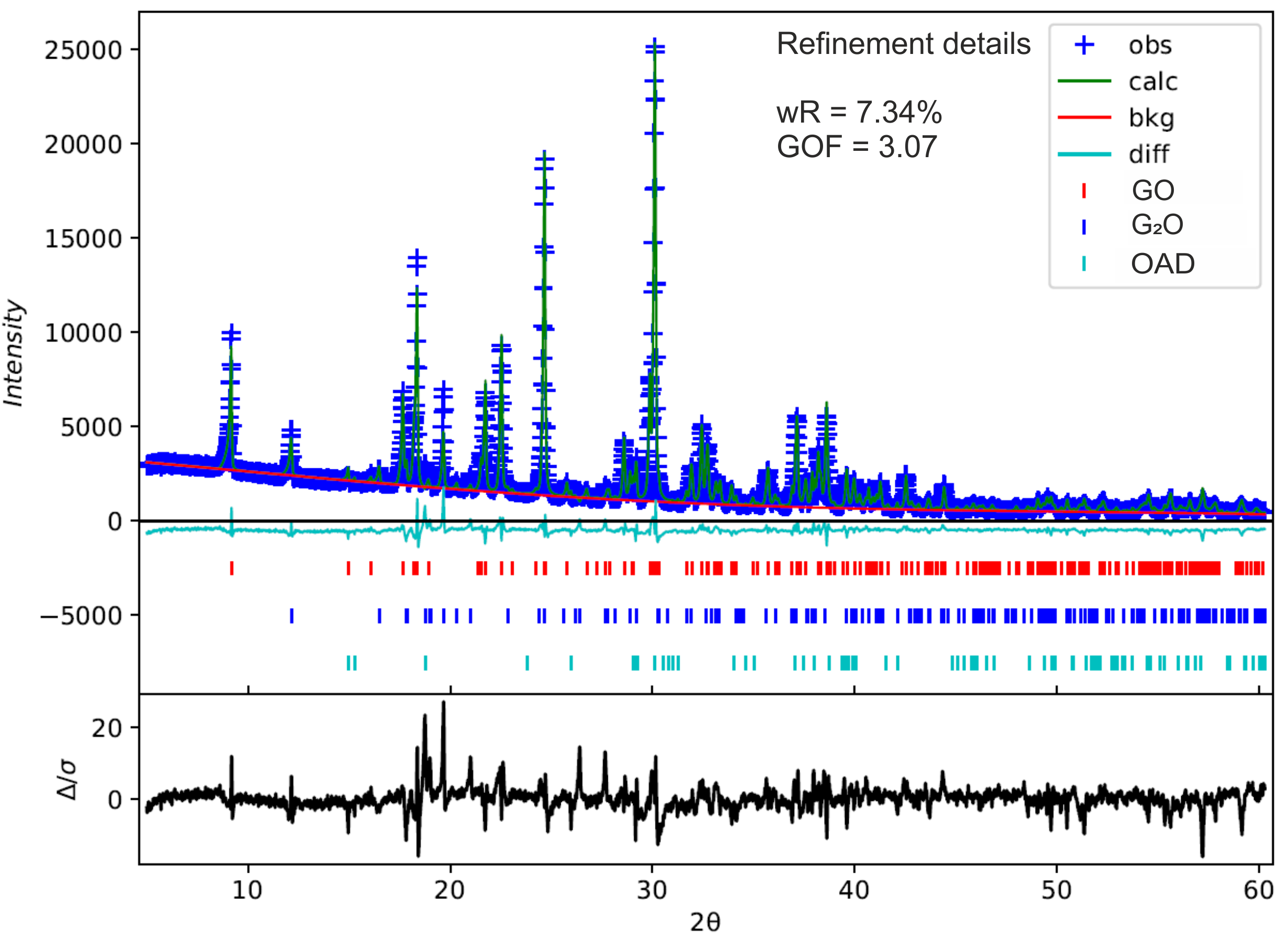


Figure S6. Profile fitting of the powder diffraction pattern: G_2_O + OAD mixture, treated for 210 s (Figure 7 in the main text)


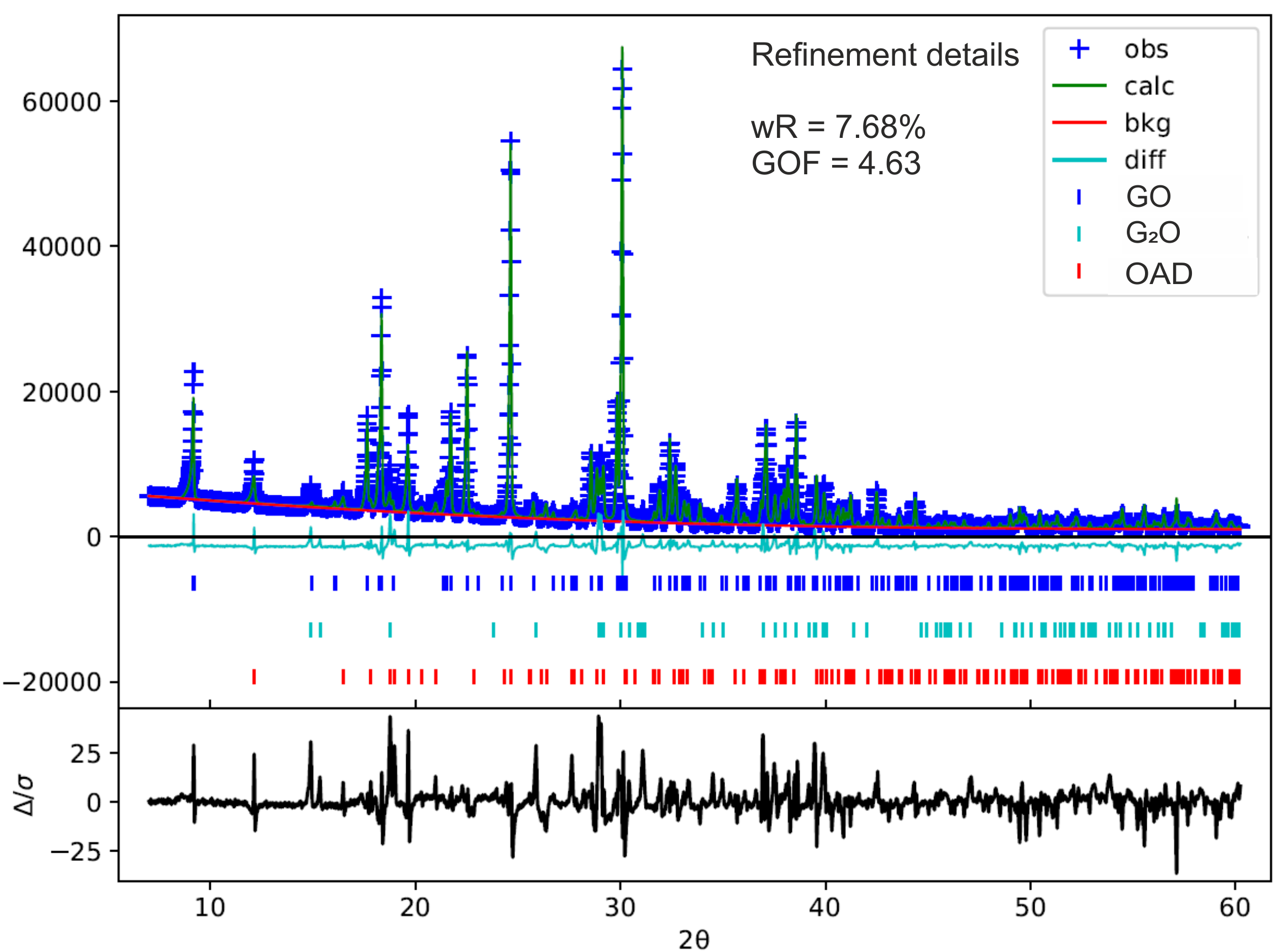


Figure S7. Profile fitting of the powder diffraction pattern: α-glycine + OAD mixture, treated for 60 s (Figure 6 (high humidity) in the main text)
